# Pharmacoperone rescue of vasopressin 2 receptor mutants reveals unexpected constitutive activity and coupling bias

**DOI:** 10.1371/journal.pone.0181830

**Published:** 2017-08-02

**Authors:** Jo Ann Janovick, Timothy P. Spicer, Thomas D. Bannister, Louis Scampavia, P. Michael Conn

**Affiliations:** 1 Departments of Internal Medicine and Cell Biology/Biochemistry, Texas Tech University Health Sciences Center, Lubbock, Texas, United States of America; 2 Lead Identification Division, Translational Research Institute and Department of Molecular Therapeutics, Scripps Research Institute, Jupiter, Florida, United States of America; 3 Department of Chemistry, Scripps Research Institute, Jupiter, Florida, United States of America; Universita degli Studi di Bari Aldo Moro, ITALY

## Abstract

Pharmacoperones are small molecules that diffuse into cells and rescue misfolded, mistrafficked protein mutants, restoring their function. These substances act with high target specificity, serving as templates to fold (or refold) receptors, enzymes, ion channels or other proteins and enable them to pass the scrutiny of the cellular quality control system (“rescue”). In the present study we demonstrate that a rescued mutant (L83Q) of the vasopressin type 2 receptor (V2R), shows a strong bias for Gs coupling unlike the WT V2 receptor, which couples to both Gs and Gq/11. Failure of the mutant to couple to Gq/11 was not due to a limiting quantity of G-proteins since other Gq/11-coupled receptors (WT V2R, histamine receptor and muscarinic receptor) responded appropriately to their ligands. Transfection with DNA encoding Gq enabled the V2 receptor mutant to couple to this G protein, but only modestly compared with the WT receptor. Fourteen V2R mutant pharmacoperones, of multiple chemical classes, obtained from a high throughput screen of a 660,000 structure library, and one V2R peptidomimetic antagonist rescues L83Q. The rescued mutant shows similar bias with all pharmacoperones identified, suggesting that the bias is intrinsic to the mutant protein’s structure, rather than due to the chemical class of the pharmacoperone. In the case of V2R mutant Y128S, rescue with a pharmacoperone revealed constitutive activity, also with bias for Gs, although both IP and cAMP were produced in response to agonist. These results suggest that particular rescued receptor mutants show functional characteristics that differ from the WT receptor; a finding that may be important to consider as pharmacoperones are developed as therapeutic agents.

## Introduction

Pharmacoperones are chemical species that rescue misrouted mutant proteins by enabling them to pass the cellular quality control system that normally prevents improperly folded proteins from being utilized [1–3]. Because the quality control system recognizes common motifs of misfolding (unpaired Cys residues, exposed hydrophobic plates) [4], rather than specific defects that interfere with function, many misfolded mutants are actually fully functional, but misrouted [5, 6].

Unrescued mutants are frequently routed back to the endoplasmic reticulum or elsewhere in the cell and never reach their biological site of action, resulting in loss-of-function diseases. Pharmacoperones are able to rescue both newly-synthesized proteins and proteins that have been returned to the endoplasmic reticulum for degradation or reprocessing [7]; accordingly these drugs can reverse the course of disease progression.

Pharmacoperones, while generally highly target-specific, can rescue multiple mutants of a specific protein [8], even though they may be widely dispersed over the protein. These characteristics are important features of this class of drugs, since accumulation of misfolded proteins in the ER stresses the cell in other ways, including evoking the unfolded protein response, which can worsen diseases or result in apoptosis [9–12].

For some pharmacoperones, the biochemical mechanism of action has been determined [13] and *in vivo* proof-of-principle has been achieved [14]. Because mistrafficked mutants do not reach their biological site of action (so coupling cannot be assessed) and the use of pharmacoperones is a relatively new approach in drug discovery, there is little information regarding the characteristics of rescued mutants, such as whether they exhibit constitutive activity or second messenger bias. The WT V2R predominantly signals through coupling to Gs, however, it has been shown to couple to Gq by activating phospholipase C [15, 16]. There are several V2R mutants that are retained in the ER, including the L83Q V2R mutant. It has been reported to accumulate in a pre-Golgi compartment rather than at the plasma membrane [17]. The Y128S V2R mutant shows partial expression at the plasma membrane but mainly resides in the ER or ER-Golgi compartment [18]. Because the V2R mutants L83Q and Y128S are disease-associated (nephrogenic diabetes insipidus) [17, 19–21] and pharmacoperone rescue is a potential therapeutic approach to this disease, we evaluated the characteristics of these two mutants, comparing them to their WT counterpart in terms of second messenger bias and constitutive activity.

## Materials and methods

### Materials

SR121463B is a V2R antagonist and known pharmacoperone that was used in the current study, after being generously provided by Dr. Claudine Serradeil at Sanofi-Aventis and used as received. Other pharmacoperones were identified by us by high throughput screening of a large chemical library [22, 23]. Several reagents were used as obtained from indicated vendors: 3-Isobutyl-1-methylxanthine (IBMX, Sigma Aldrich, St. Louis, MO), vasopressin (Tocris Biosciences, Bristol, England UK), fetal calf serum (FCS, Hyclone, Logan, UT), Dulbecco’s MEM (DMEM), PBS (GIBCO, Invitrogen). pTRE2-Hygromycin vector (Invitrogen, San Diego, CA), human arginine-vasopressin 2 receptor (V2R; Gene Bank Accession Number: AY242131), Gq plasmid (Gene Bank Accession Number: U43083) [24]; both plasmids from cDNA Resource Center; www.cdna.org;), myo-[2-^3^H(N)]-inositol (NET-114A; PerkinElmer, Waltham, MA), vasopressin (8-L-arginine), [phenylalaninyl-3,4,5-^3^H(N)- (NET800, specific activity = 66.3 Ci/mmol; PerkinElmer, Waltham, MA), and unbound 125-Iodine (016303710; MP Biomedicals, Santa Ana, CA).

### Creation of mutant receptors

Mutants L83Q (CTG → CAA) and Y128S (TAC →AGT) V2R cDNAs for transfection were prepared by us using overlap extension PCR [25]. The L83Q mutant was cloned into both pcDNA3.1 (for transient transfection) and pTRE2-Hygromycin (for stable transfection) vectors and the Y128S mutant was cloned into pcDNA3.1 vector. The restriction enzymes used for pcDNA3.1 are EcoRI and XhoI. The restriction enzymes used for pTRE2-Hygromycin are BamHI and ClaI. The purity and identity of plasmid DNAs were verified by dye terminator cycle sequencing (Applied Biosystems, Foster City, CA).

### Creation and use of stable (tTA + L83Q mutant and hWT V2R receptors) HeLa cells

The stable HeLa (tTA; tetracycline-controlled transactivator) cell line [26] was a kind gift from Dr. Peter Seeburg (he passed away on August 22, 2016; Max Planck Institute for Medical Research in Heidelberg, Germany). The cells were maintained in growth medium, DMEM, /10% FCS/20 μg/ml gentamicin) and grown at 37°C, 5% CO_2_ in a humidified atmosphere until the density reached about 90%.

The human WT V2R and the mutant L83Q were cloned into pTRE2-hygromycin vector (the response vector) and then transfected into the stable HeLa cell line (tTA; tTA binds the TRE and activates transcription in the absence of tetracycline or doxycycline). Selection antibiotics were used at 400 μg/ml G418 + 200 μg/ml hygromycin. Single colonies were selected and screened for expression of the WT V2R and mutant L83Q receptors in separate, stably transfected cells. All cell lines were tested and treated for mycoplasma; they are all negative for mycoplasma prior to performing all of the experiments.

Fifty thousand cells of the stable HeLa line containing tTA for transient transfections or the stable HeLa cells (containing tTA + hWT V2R or the mutant L83Q) were plated in 48-well Costar cell culture plates and transiently transfected or co-transfected with100 ng total cDNA (per 0.125 ml) of hWT V2R, L83Q, Y128S mutants, Gq unless otherwise indicated. Cells were cultured in growth medium DMEM, 10% fetal calf serum (FCS), and 20 μg/ml gentamicin] at 37°C in a 5% CO2 humidified atmosphere. Twenty-four hours after plating, the cells were washed with 0.5 ml of OPTI-MEM and then transfected with WT or mutant receptor DNA with pcDNA3.1 (empty vector) to keep the total DNA constant (100 ng/ 0.125 ml). Lipofectamine was used according to the manufacturer's instructions. Five hours after transfection, 0.125 ml of DMEM with 20% FCS and 20 μg/ml gentamicin was added. Twenty-three hours after transfection, the medium was replaced with 0.25 ml of fresh growth medium. Where indicated, pharmacoperones (10 μM for all except 1 μM for compound #50) in 1% DMSO (vehicle) were added for 16–18 h in respective media to the cells and then removed before agonist treatment. Cyclic AMP release and IP production was measured as described below.

### Inositol phosphate (IP) determination

Cells were washed twice with DBG (DMEM/0.1% BSA/20 μg/ml gentamicin) then “preloaded” with 4 μCi/ml myo-[2-^3^H(N)]-inositol in inositol-free DMEM containing 10 μM pharmacoperone or 1% DMSO as a control (final concentration) in quadruplicate and allowed to incubate for 18 h. After the preload, the cells were washed in 0.3 ml/well for 10 min at 37°C twice then once for 20 min at 37°C with DMEM (inositol free) containing 5 mM LiCl + 1% DMSO and then treated for 2 h with 1 μM vasopressin in the same medium or 30 min with histamine dihydrochloride or acetylcholine, times which provide optimal response. Total IP was then determined by chromatographic separation as previously described [27].

### cAMP determination

After 16 h, the cells were washed in 0.5 ml/well with DBG containing 1% DMSO to wash out the pharmacoperones. The washed cells were incubated for 10 min at 37°C twice, then once for 20 min at 37°C. The cells were then stimulated with 1 μM vasopressin, histamine dihydrochloride or acetylcholine in DBG containing 0.2 mM 3-Isobutyl-1-methylxanthine (IBMX; to prevent degradation of cAMP) for 30 min at 37°C. After stimulation, the medium from each well was collected in glass test tubes containing 10 mM theophylline (final 1 mM). The samples were heated at 99°C for 5 min and RIA for cAMP was determined. cAMP accumulation (extracellular) was measured in acetylated samples by RIA as previously described [28]. cAMP antiserum R3B5a (prepared in our laboratory) [29] was used at a titer of 1:5000; this antiserum showed less than 0.1% cross-reactivity with cGMP, 2′,3′-cAMP, 5′-cAMP, 3′-cAMP, ADP, GDP, ATP, CTP, and 3-Isobutyl-1-methylxanthine. All cAMP data in this paper have not been published before, they are new experiments done independently.

### Scatchard assay

Stably transfected HeLa cells containing the tTA plus human V2R WT or mutant (L83Q) were cultured and plated in growth medium at 100,000 cells per well in 24-well Costar cell culture plates. Fifty two hours after plating, the cells were washed twice with DMEM/ 0.1% BSA/ Gentamicin and 1 μM pharmacoperone or DMSO was added and allowed to incubate for 17 hours at 37°C. The cells were then washed twice for 10 minutes at 37°C, then 1, 20 minute wash at 37°C with DMEM/ 0.1% BSA/ Gentamicin with 1% DMSO to wash out the pharmacoperone. Increasing concentrations of [^3^H]-AVP (1.56–50 nM; specific activity = 66.3 Ci/mmol) was added to the cells in DMEM/ 0.1% BSA/ 10 mM HEPES medium and allowed to incubate at room temperature for 60 min, consonant with maximum binding [30]. New receptor synthesis during this period is negligible at room temperature. After 60 min, the media was removed, cells were washed quickly with PBS twice, 0.2 N NaOH was added to the wells, liquid was transferred to scintillation vials and radioactivity was measured using a Beckman LS 6500 scintillation counter. To determine nonspecific binding, the same concentration of radioligand was added to cells in the presence of 4.6 μM unlabeled vasopressin.

### RNA extraction for real-time PCR

HeLa cells containing tTA, hWT V2R or L83Q V2R mutant were plated in a 6-well culture plate at 2 × 10^5^ cells per well. Trizol Reagent was added to the cells for extracting total RNA and processed according to the manufacturer’s instructions. Reverse transcription was performed using 1 μg total RNA and SuperScript III Reverse Transcriptase (Invitrogen) for converting RNA to cDNA according to the supplier’s protocol.

### Real-time PCR

Applied Biosystems^™^ TaqMan^™^ Assays for detecting the human V2R, V1a, V1b, and Oxytocin receptor were used (Applied Biosystems, Foster City, CA). The Applied Biosystems^™^ QuantStudio^™^ 12K Flex system and reagents were used for generating the real-time PCR data. 18s RNA was used as the endogenous control and ΔCt was used to indicated if the gene was present. Samples were run in triplicate in at least three separate experiments (n = 3 ± SEM).

### High throughput screening (HTS) compounds

Test compounds were obtained from commercial vendors and passed purity evaluation (>95% pure) based upon LCMS analysis. Compounds were selected for purchase based upon structural similarity to known active compounds, based upon Tanimoto score and medicinal chemist’s judgement regarding tractability. Compounds reported here include: N-(3-(thiazolo[5,4-b]pyridin-2-yl)phenyl)-1-naphthamide (**4**), N-(4-(7-ethoxybenzofuran-2-yl)thiazol-2-yl)cyclopropanecarboxamide (**17**), N-benzyl-3-chloro-N-phenyl-5-(p-tolyl)-7-(trifluoromethyl)-4,5,6,7-tetrahydropyrazolo[1,5-a]pyrimidine-2-carboxamide (**22**), N-(3-(2-((5-chloropyridin-2-yl)amino)thiazol-4-yl)phenyl)acetamides (**32**), (1s,3s)-N-(4,6-dimethylbenzo[d]thiazol-2-yl)adamantane-1-carboxamide (**50**), N-(4-(2,3-dihydrobenzo[b][1,4]dioxin-6-yl)thiazol-2-yl)-2-(2,4-dimethylphenyl)acetamides (**54**), N-(3,5-dichlorophenyl)-4-fluoro-3-(N-(2-methoxybenzyl)sulfamoyl)benzamide (**68**), 4-(N,N-diethylsulfamoyl)-N-(4-(naphthalen-2-yl)thiazol-2-yl)benzamide (**73**), 2,5-dimethyl-N-(4-(methylthio)benzo[d]thiazol-2-yl)furan-3-carboxamide (**78**), N-(3-(2-((3-chloro-2-methylphenyl)amino)thiazol-4-yl)phenyl)benzamide (**81**), 4-(N,N-dipropylsulfamoyl)-N-(4-methoxybenzo[d]thiazol-2-yl)benzamide (**84**), 2-(benzo[d][1,3]dioxol-5-yl)-N-(4-ethylbenzo[d]thiazol-2-yl)acetamides (**89**), 2-oxo-N6,N8-di-m-tolyl-1,2-dihydrobenzo[cd]indole-6,8-disulfonamide (**93**), and cyclohexyl 2-methyl-5-oxo-7-phenyl-4-(p-tolyl)-1,4,5,6,7,8-hexahydroquinoline-3-carboxylate (**94**). The numbers in bold and underlined were assigned this number for the studies conducted in this manuscript.

### Statistical analysis

Data (>3) were analyzed with one-way analysis of variance and then paired student’s t- test (SigmaStat 3.1; Jandel Scientific Software); *P < 0.05 was considered significant.

## Results

**Fig 1:**
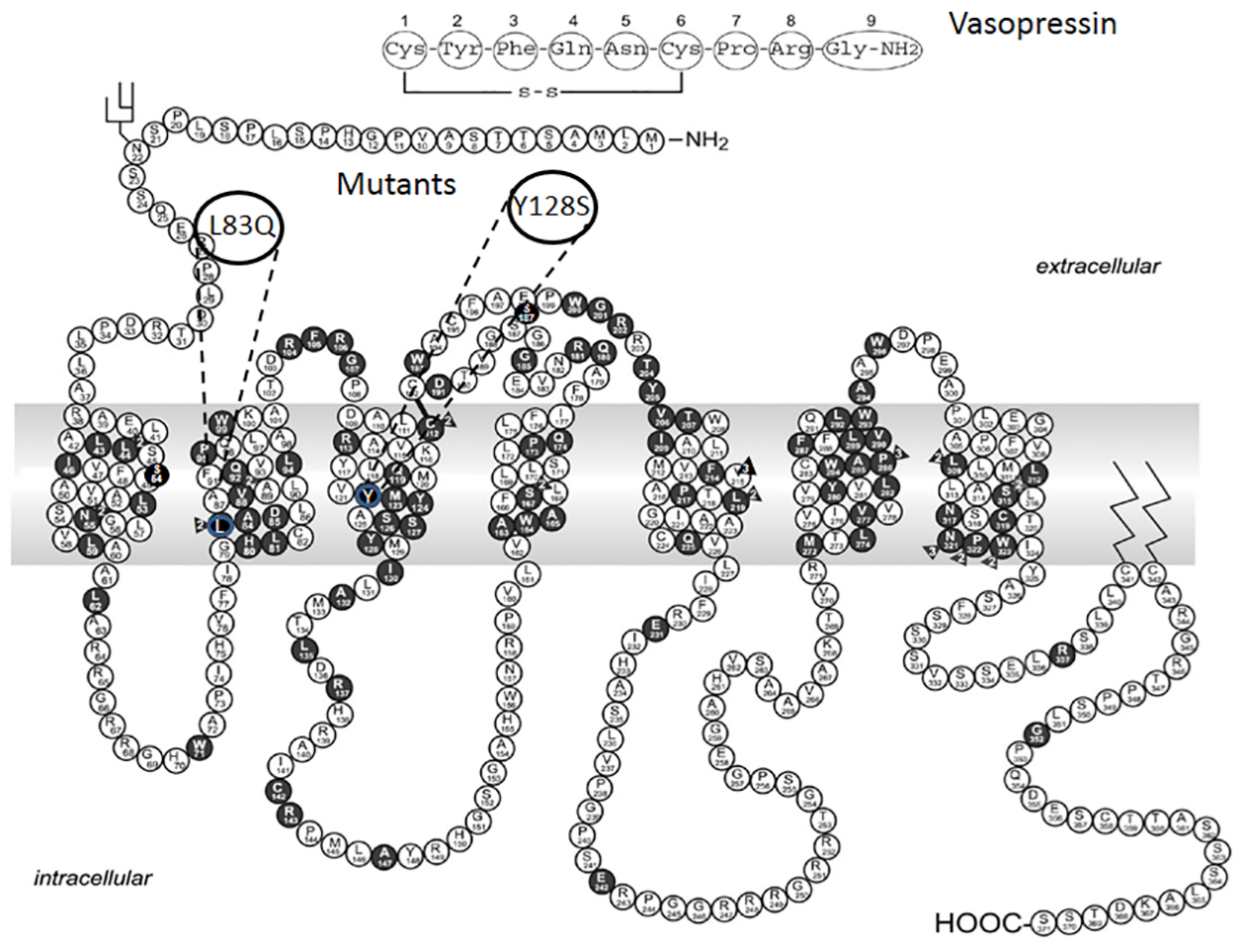
Map of the human arginine-vasopressin 2 receptor showing mutants associated with nephrogenic diabetes insipidus in black. The location of L83Q and Y128S, the mutants used in the present study, are shown. The amino acid sequence of the naturally-occurring ligand, vasopressin, is shown at the top. When there are multiple mutations at a single site, the number of mutations is noted in a triangle. The mutants in this study are a blue circle so they stand out. Adapted and modified from [36].

Fig 1 shows the location of the mutants evaluated in the present work. L83Q and Y128S are in the membrane component of the second and third transmembrane segments, respectively. These mutants are associated with nephrogenic diabetes insipidus. Amino acid changes in these sites would be expected to impact on the first and second intracellular loops; sites believed to be involved in coupling of GPCRs to G proteins [31–35].

**Fig 2:**
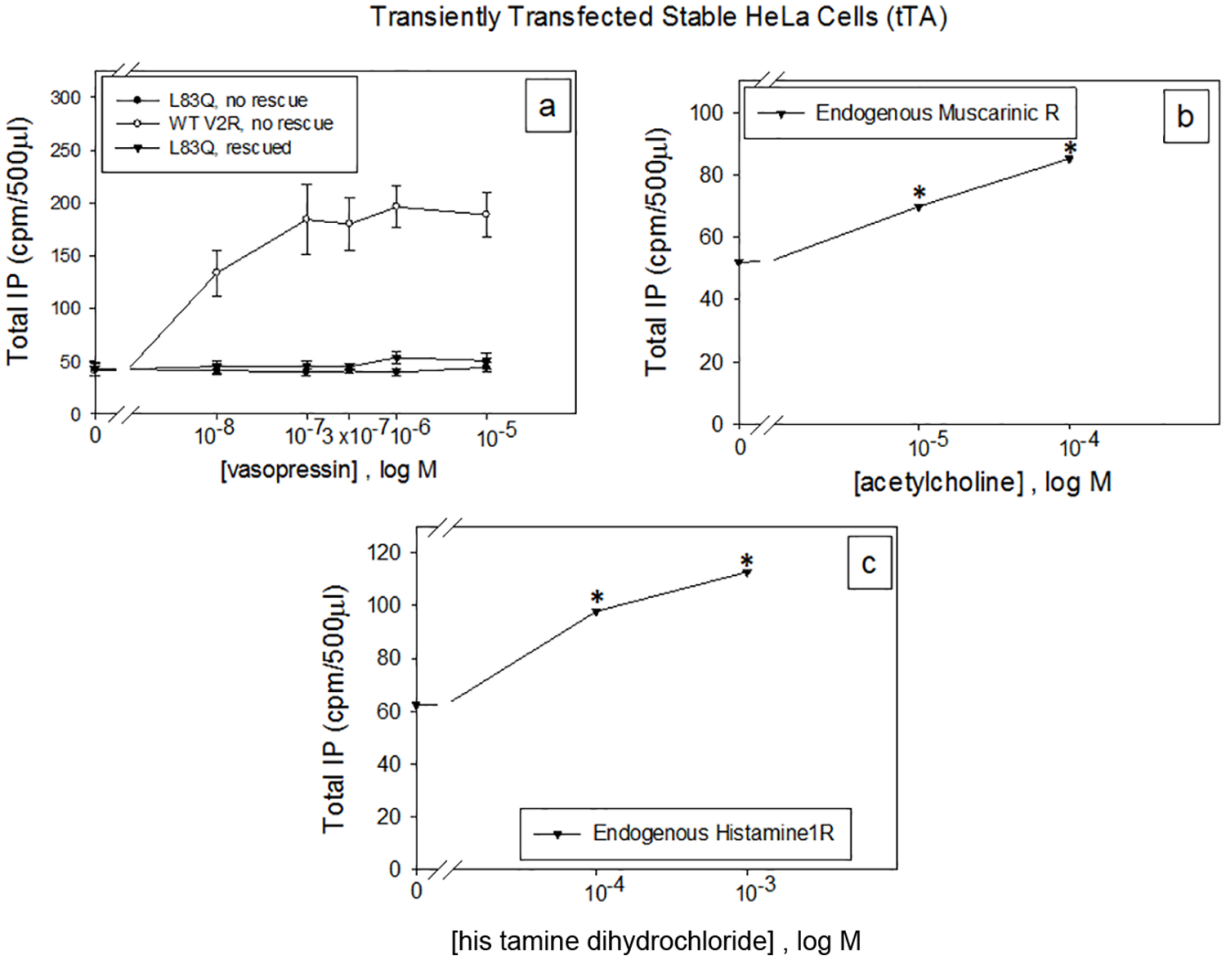
IP production following transient transfection of HeLa cells for selected GPCRs and V2R mutants. Transiently transfected HeLa cells containing a total of 100 ng cDNA/ 0.125 ml of hWT V2R or the mutant hL83Q were used to compare Gq (IP) coupling with the vasopressin receptor, endogenous muscarinic M3 subtype and histamine H1 receptors with or without 10 μM SR121463B pharmacoperone (rescues the L83Q mutant), DMSO (vehicle) is a negative control. Cells were stimulated with various doses of vasopressin for 2 h, acetylcholine and histamine dihydrochloride for 30 minutes. The results shown in the figure are from at least 3 independent experiments performed in quadruplicate (n = 3 ± SEM), p value < 0.05 is considered significant.

**Fig 3:**
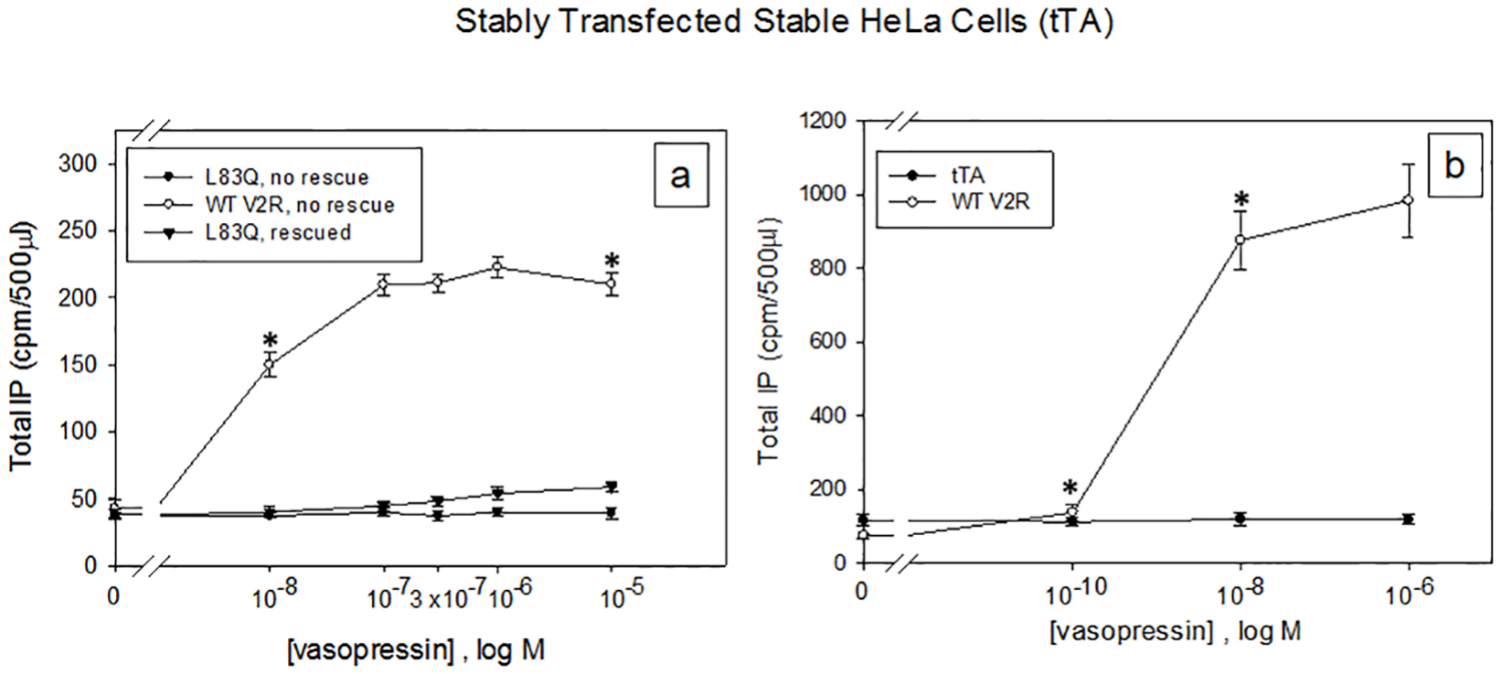
IP production from HeLa cells stably expressing WT V2R, mutant L83Q or tTA. Stably transfected HeLa cells containing tTA + hWTV2R or tTA + L83Q mutant were used to compare Gq (IP) coupling (a) with the vasopressin receptor with or without 10 μM SR121463B pharmacoperone (rescues the L83Q mutant), DMSO (vehicle) is a negative control. (b) Stably transfected HeLa cells with the WT V2R or tTA alone, were used to show if the oxytocin, V1a or V1b receptors are being stimulated with vasopressin to produce IP. Cells were stimulated with various doses of vasopressin for 2 h because the HeLa cells produce low amounts of total IP with the addition of 5 mM LiCl. The results shown in the figure are from at least 3 independent experiments performed in quadruplicate (n = 3 ± SEM), p value < 0.05 is considered significant.

Figs 2 and 3 show that WT vasopressin receptor (a in both figures) in transiently (Fig 2) and stably transfected cells (Fig 3) respectively, couples to Gq for IP production, but the mutant L83Q (a, rescued or not) does not couple to the IP pathway (a) although the endogenous muscarinic M3 subtype (2b) [37, 38] and histamine H1 (2c) [39–41] receptors, that are normally found in HeLa cells, respond to their respective agonists with IP production. Fig 3b shows that the HeLa cells stably transfected with just the tTA (tetracycline-controlled transactivator), there is no IP response to vasopressin which indicates that the oxytocin, V1a or V1b receptors, which couple to Gq, are not responding to the vasopressin [35, 42]. Note that whether using the transient or stable system the response to all conditions is the same. This observation seen with the acetylcholine and histamine receptors suggests that there is adequate G protein found in these cells to enable those receptors, as well as for the WT V2R, to couple. Real-Time PCR using the TaqMan assay with 18s RNA as the endogenous control and measuring the ΔCt, indicates that the HeLa cells used in all of the experiments do not contain the oxytocin, V1a, or V1b receptors because there was no amplification of any of these 3 receptors using 100 ng cDNA. There was amplification of the 18s RNA using 100 ng cDNA (ΔCt = 27.74 ± 0.002), 100 ng cDNA WT V2R (ΔCt = 30.00 ± 0.003) and 100 ng cDNA L83Q mutant (ΔCt = 31.14 ± 0.43).

**Fig 4:**
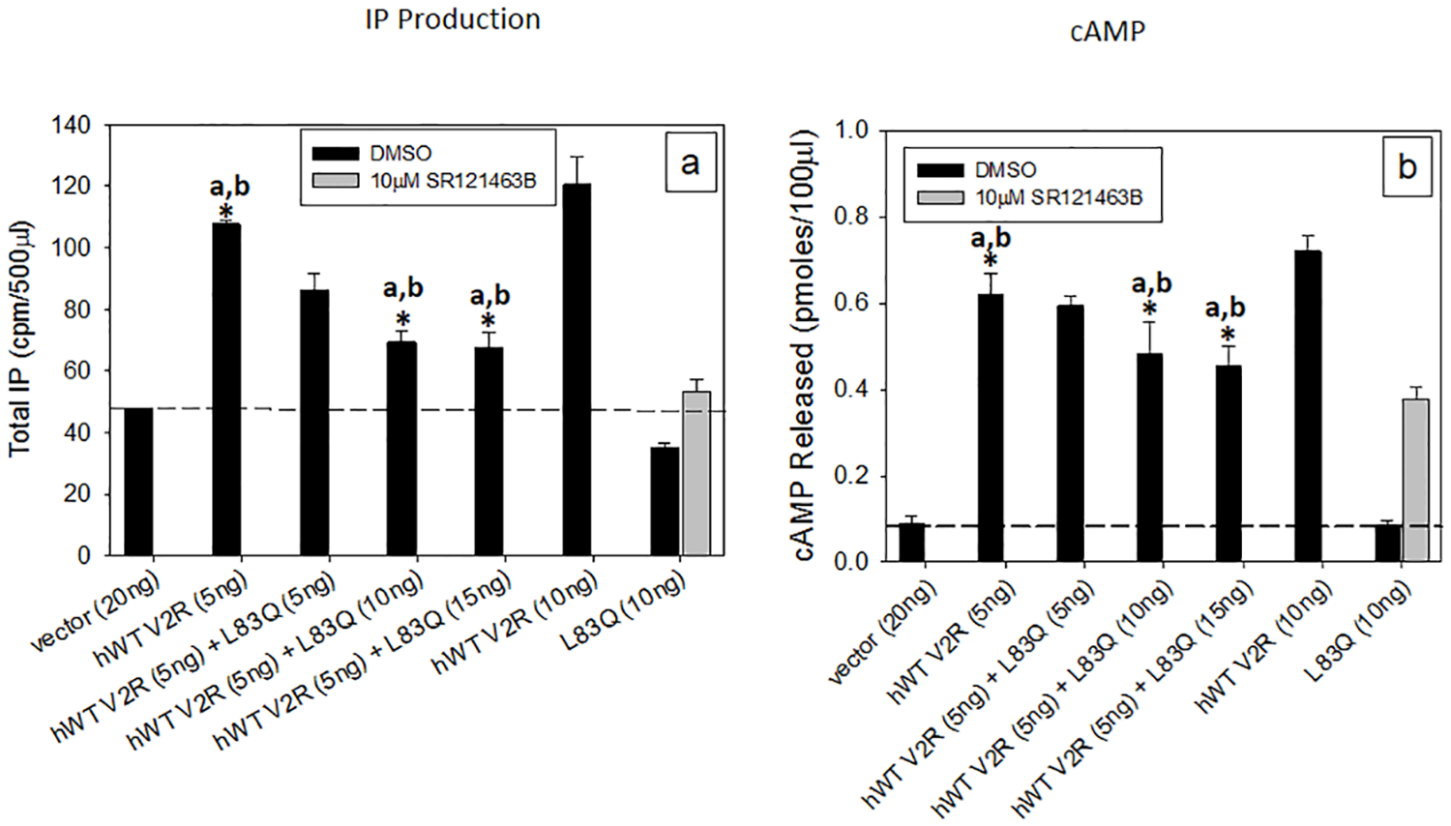
IP or cAMP production from HeLa cells transiently transfected with WT V2R in the presence of increasing amounts of mutant. Cells were transiently transfected with an increasing amount of cDNA for the L83Q mutant in the presence of hWT V2R, keeping the WT at 5 ng and keeping the total amount of cDNA at 20 ng by supplementing the difference with empty vector pcDNA3.1. The cells were stimulated with 1 μM vasopressin and IP production (a) or cAMP (b) was measured. The dashed line shows the response to the empty vector pcDNA3.1. The results shown in the figure are from at least 3 independent experiments performed in quadruplicate (n = 3 ± SEM), p values < 0.05 is considered significant. *a, p < 0.05 compared with vector and WT V2R IP or cAMP production, *b, p < 0.05 compared with WT V2R and WT V2R + L83Q (10 and 15ng) IP or cAMP production.

Fig 4 shows transiently transfected HeLa cells expressing WT V2R alone, the mutant L83Q alone, or both together. When L83Q alone was present, cells were either treated with SR121463B (rescued) or not. The data show that the WT receptor, couples to both Gq (4a) and Gs (4b), but the mutant only couples to Gs (4a, b). When both are present in the absence of rescue, the mutant shows a dominant negative effect on the WT receptor. This effect has been reported for other GPCR mutants and their corresponding WT receptor [43–51]. This effect appears to be the result of WT-mutant oligomers being transferred to the plasma membrane, but failing the quality control system when combined [52, 53]. In the case of the gonadotropin releasing hormone receptor and its mutants, it appears that mutants retain WT in the endoplasmic reticulum [48].

**Fig 5:**
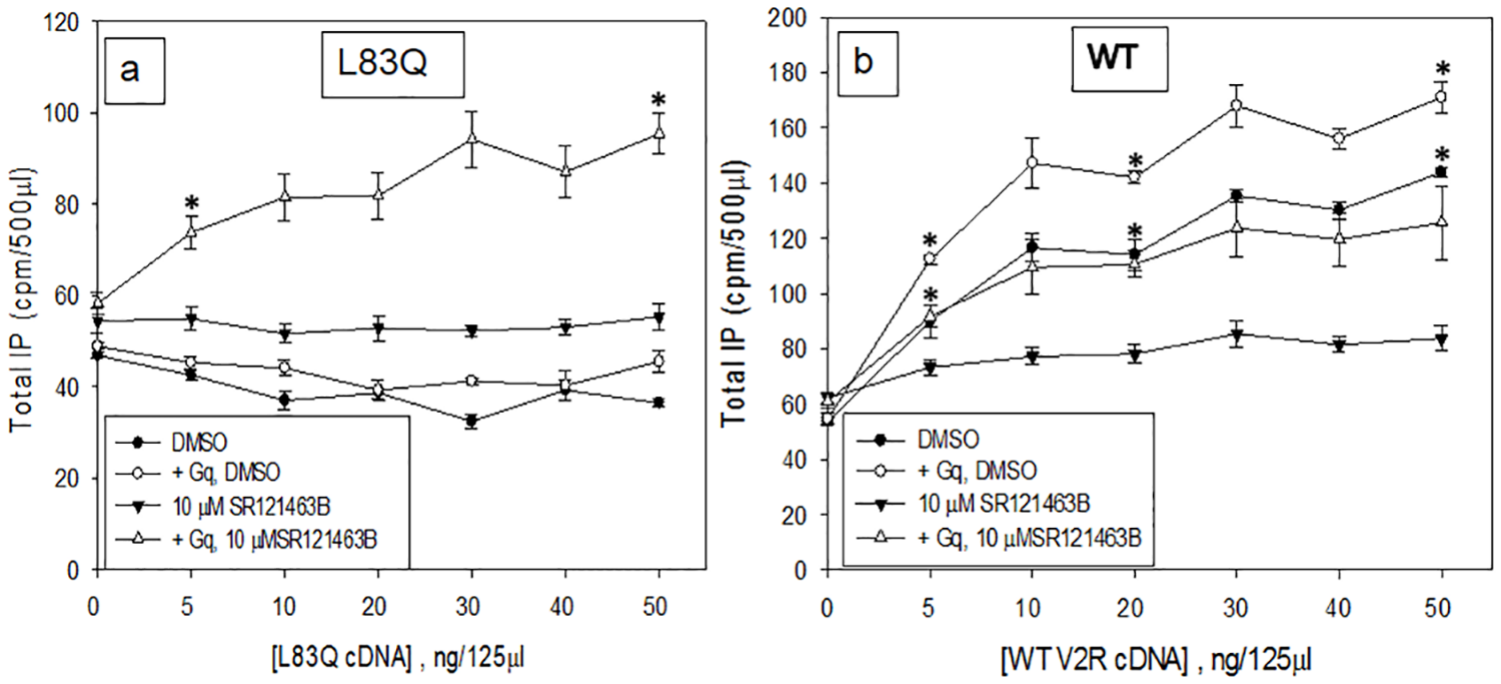
IP production by HeLa cells, with or without cDNA for Gq, with or without rescue by pharmacoperone SR121463B. HeLa cells were transiently co-transfected with L83Q mutant + 10ng Gq (a) or hWT V2R + 10ng Gq (b) to assess IP response with with or without rescue. Cells were either treated with SR121463B or not (in which case an equivalent amount of DMSO was present). Cells were stimulated with 1 μM vasopressin for 2 hours. The results shown in the figure are from at least 3 independent experiments performed in quadruplicate (n = 3 ± SEM), p values < 0.05 is considered significant.

In a separate study (Fig 5), HeLa cells were transiently transfected with increasing amounts of L83Q mutant or WT V2R while keeping the Gq cDNA constant at 10 ng because it has been reported that HeLa cells contain only small amounts of Gq [54]. We chose not to transfect G11 because the HeLa cells contain sufficient amounts of endogenous G11 [54]. When the mutant L83Q was rescued with pharmacoperone SR121463B (a) in Gq-supplemented cells, a small amount of coupling could be seen, but less than half of what is observed with WT receptor (b). This activity is likely due to pushing the mutant plus Gq → mutant-Gq far to the right and shows that mutants with poor ability to bind Gq can couple to Gq in the presence of large amounts of the mutant or WT protein, if only modestly.

**Fig 6:**
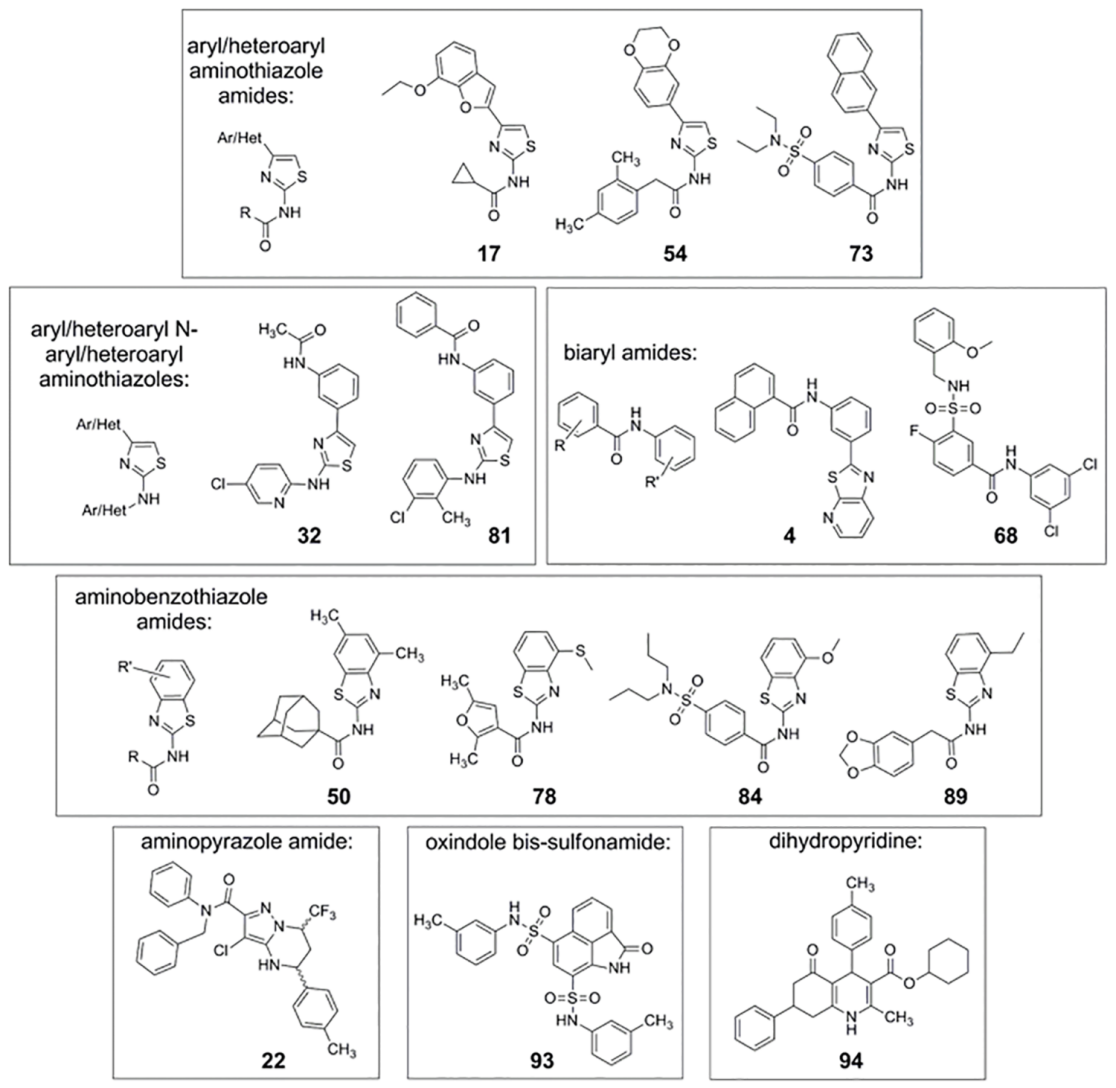
Pharmacoperones used in this study, showing that compounds in many different structural classes rescue the mutant L83Q. The 14 compounds shown have been grouped into 7 structure classes.

**Fig 7:**
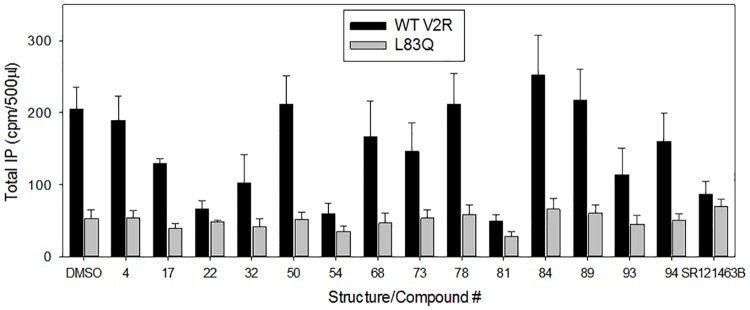
Rescue of IP production by WT and mutant-expressing HeLa cells by various pharmacoperones. Transiently transfected HeLa cells containing a total of 100 ng cDNA / 0.125 ml of hWT V2R or the L83Q mutant were treated with different pharmacoperones (10 μM, or #50 utilized at 1 μM) to show if the rescued L83Q mutant is coupled to Gq (IP). The cells were incubated with the pharmacoperones for 18 h with 4 μCi/ml ^3^H-inositol for “preloading” IP pathway, then washed and stimulated with 1 μM vasopressin for 2 h and IP response was measured. DMSO (vehicle) is a negative control that does not rescue the mutant and SR121463B is a known pharmacoperone for the L83Q mutant. The results shown in the figure are from at least 3 independent experiments performed in quadruplicate (n = 3 ± SEM).

**Fig 8:**
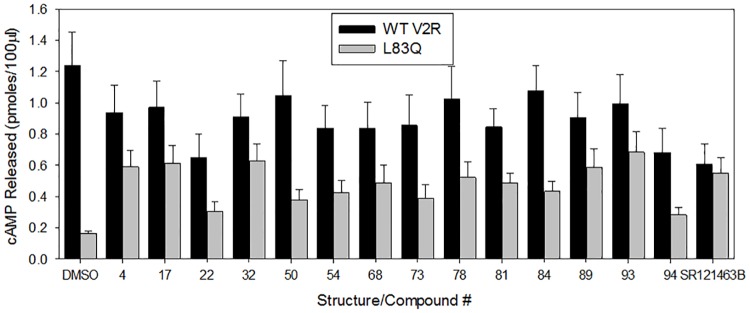
Rescue of cAMP production by WT and mutant-expressing HeLa cells by various pharmacoperones. Transiently transfected HeLa cells containing a total of 100 ng cDNA / 0.125 ml of hWT V2R or the L83Q mutant were treated with different pharmacoperones (10 μM or #50 was 1 μM) to show if the rescued L83Q mutant is coupled to Gs (cAMP). The cells were incubated with the pharmacoperones for 16 h, then washed and stimulated with 1 μM vasopressin containing 0.2 mM IBMX for 30 minutes and the cAMP response was measured. DMSO (vehicle) is a negative control that does not rescue the mutant and SR121463B is a known pharmacoperone for the L83Q mutant. The results shown in the figure are from at least 3 independent experiments performed in quadruplicate (n = 3 ± SEM).

Because our HTS effort identified many different chemical structures that are capable of serving as pharmacoperones, we used many different compounds (Fig 6) to treat cells expressing WT V2R or the mutant L83Q. We showed that none of these chemical structures enable coupling of the mutant to IP, although the WT V2R coupled (Fig 7); while virtually all test compounds allowed the mutant to couple to cAMP (Fig 8). Note, SR121463B was included as the control ligand/ pharmacoperone in each experiment and elicited the appropriate response.

**Fig 9:**
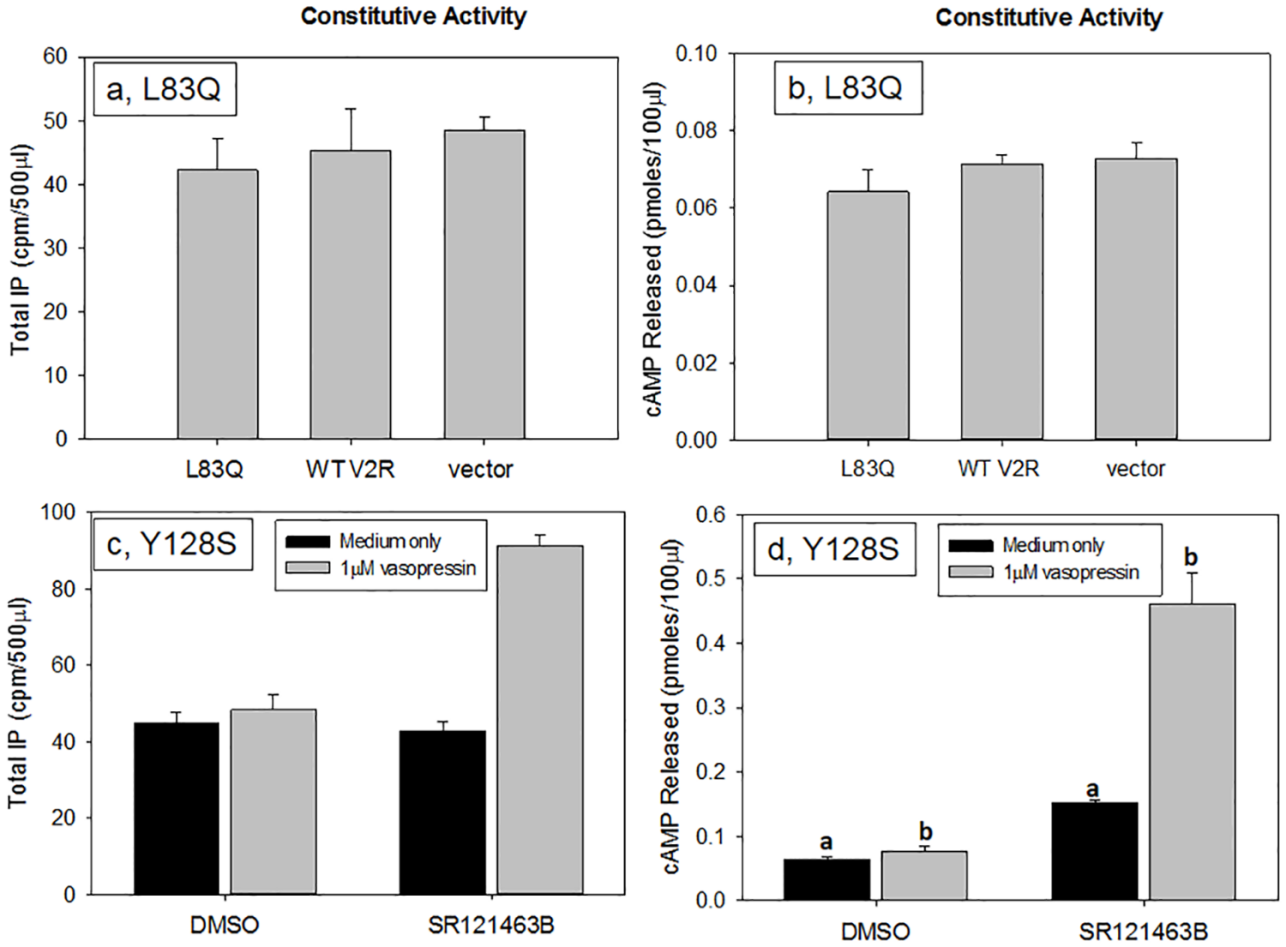
Constitutive or stimulated activity of V2R mutants with or without rescue. Transiently transfected HeLa cells containing a total of 100ng cDNA / 0.125 ml of hWT V2R, L83Q mutant and empty vector were used to assess if there is constitutive activity (CA) with the IP pathway, 9a, or with the cAMP pathway, 9b. Figure 9c shows that Y128S, is coupled to Gq/11 in the presence of agonist (1 μM vasopressin for 2 hours), but does not show constitutive activity (no agonist). Figure 9d shows constitutive activity of Y128S for the cAMP pathway. Cells were stimulated for 30 minutes with 1 μM vasopressin. The results shown in the figure are from at least 3 independent experiments performed in quadruplicate (n = 3 ± SEM), p values < 0.05 is considered significant.

Fig 9 shows that, following rescue with SR121463B, L83Q does not constitutively activate either IP (a) or cAMP (b), rescued Y128S does not show constitutive activity for activation of IP (c) but it does for cAMP when the receptor is rescued and stimulated with media only (d), suggesting that bias may occur even in the case of constitutive activity.

**Fig 10:**
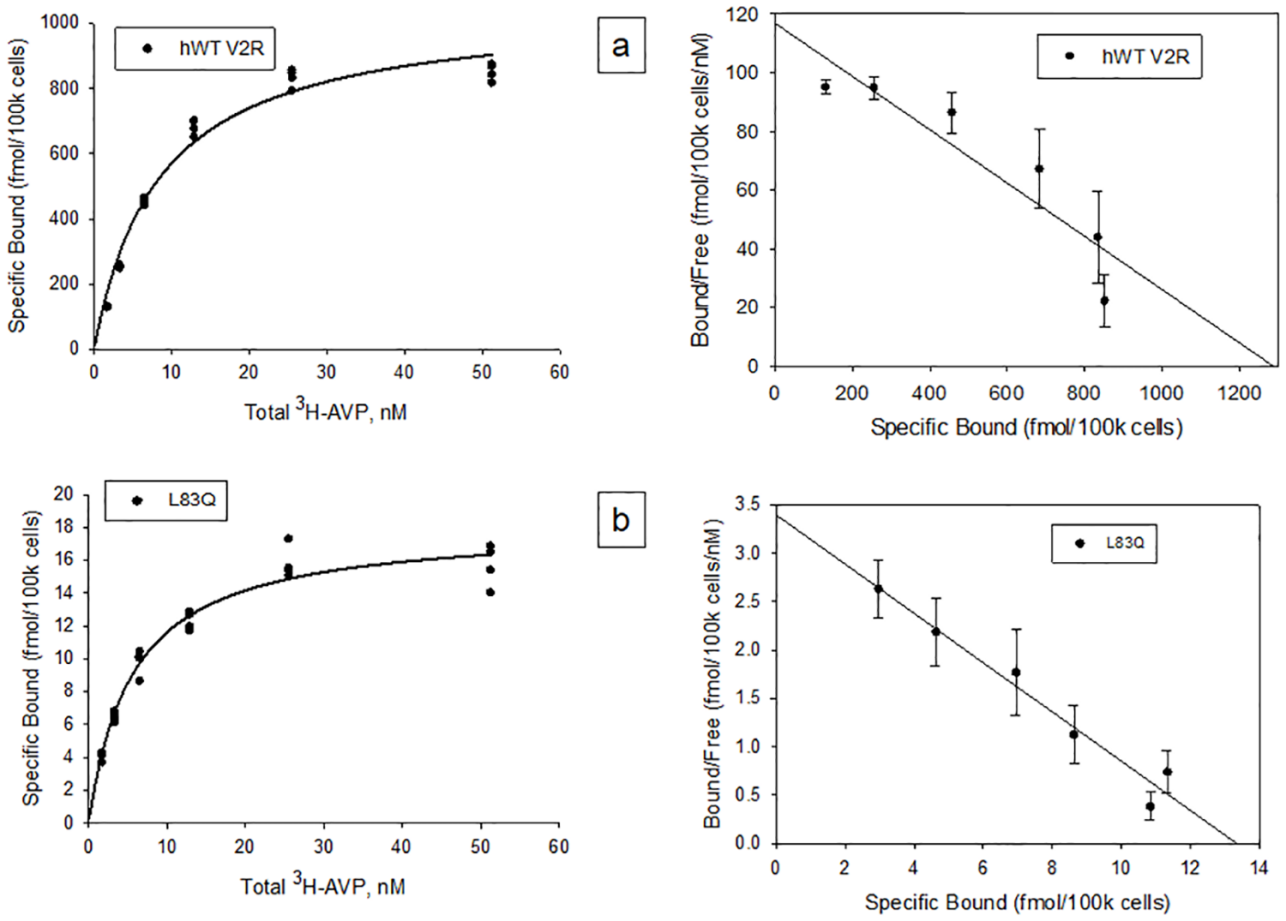
Scatchard assay of stably transfected HeLa cells with WT V2R or mutant. Stably transfected HeLa cells containing tTA + hWT V2R (a) or tTA + L83Q mutants (b) were used to determine the number of receptors present at the membrane using the scatchard assay. Six concentrations of ^3^H-AVP (specific activity = 66.3 Ci/mmol) was used ranging from 1.56 to 50 nM with serial dilutions of 1:2. The averaged receptors per cell, B_max_ and K_d_ for each of the 3 experiments is shown in the graph for the mutant and the WT V2R.

Fig 10 shows the Scatchard assay of stably transfected HeLa cells with the WT V2R (a) or the L83Q mutant (b) after the mutant was rescued with SR121463B. The number of receptors per cell, K_d_, B_max_ was determined from at least 3 independent experiments and averaged. The WT V2R had a high number of receptors per cell (6,336,196) while the L83Q mutant had about 60 fold fewer receptors per cell (109,016). The B_max_ (fmol/100k cells) and K_d_ (pM) for the WT V2R was 1052 ± 70.9 and 8.4 ± 1.7 respectively. The B_max_ (fmol/100k cells) and K_d_ (pM) for the L83Q mutant was 18.1 ± 0.7 and 5.6 ± 0.7 respectively.

## Discussion

The present study shows that two rescued mutants of the human vasopressin 2 receptor behave in a non-identical fashion compared with the WT receptor in terms of constitutive activity and effector coupling. While the WT V2R activates both the IP and cAMP pathways when stimulated by vasopressin, the rescued mutant L83Q shows a strong bias for the cAMP pathway, but no constitutive activity. Failure of the mutant to couple to Gq was not due to limiting quantities of G-protein since other endogenous receptors (muscarinic M3 subtype and histamine H1 receptors) responded appropriately to their ligands. Transient transfection with Gq cDNA enabled the V2R mutant to couple modestly to this G-protein compared to the WT V2R. While the number of receptors per cell at the plasma membrane for the WT V2R and L83Q mutant are 100 fold different in these studies, the binding affinities are similar. It has previously been reported that the mutants are expressed at lower levels than the WT due to interference with protein synthesis [55]. Transient transfection is 30% efficient which may indicate lower receptors per cell but could also be due to lower copy number per colony in the stably transfected cells containing the mutant compared to the stably transfected wild type V2R.

Evaluation of 14 chemically dissimilar compounds identified from a previous high throughput screen [22], showed a similar pattern of bias, an observation that suggested that pharmacoperones of different chemical classes may interact so as to produce a similar structural change. Such a circumstance appears to be the case of the GnRH receptor and pharmacoperones that rescue one mutant, appear to rescue most mutants, except for those that are grossly misshapen [8, 13].

The dominant negative effect shown with co-expressing the L83Q mutant and wild type V2R in the absence of rescue, appears to be the result of WT-mutant oligomers forming, failing the quality control system and remaining in the ER. Based on an early study, when truncated V2R mutants were co-expressed with the wild type V2R, complexes formed, dimerized and were determined to be retained intracellularly [56].

The Y128S mutant shows constitutive activity for the cAMP pathway after it is rescued but can activate both cAMP and IP second messenger pathways in the presence of vasopressin. Unlike the mutant Y128S, another V2R mutant, L312S, shows constitutive activity for the cAMP pathway but not the IP pathway [57]. Studies with a myc-tagged Y128S mutant was shown to be expressed but is not localized at the cell surface, however, it is localized in the ER and has defective membrane trafficking [19, 21, 58]. Y128S is not the first rescued mutant to show constitutive activity, unlike its corresponding WT molecule. Rescued gonadotropin releasing hormone receptor mutant E90K has also been shown to have constitutive activity [59, 60] and shows a different ligand specificity. The latter is not surprising, given that the site of the mutation is very near the ligand binding site. Normally E90 forms a salt bridge with K121 [61, 62]; the mutant E90K cannot form this salt bridge, of course, and would be expected to repel and distort the receptor near the site of ligand binding, potentially mimicking the effect of the binding of the ligand and constitutive activity.

Some WT receptors, such as the melanocortin 4 receptor (MC4R), show constitutive activity and biased signaling when bound by inverse agonists [63]. Laboratory prepared mutants of the MC4R, as in the case of mutants of the V2R, show bias. Ascribing a physiological role to the observation of bias in a mutant is difficult since mutants are not typically present in a healthy physiological setting. Nonetheless, the observation of these activities show the complexity possible with these signaling systems.

These findings are of interest since the ultimate use of pharmacoperones is the discovery of drugs capable of rescuing disease-causing mutants and restoring them to function. Since rescued mutants may show unexpected constitutive activity or G-protein bias, it will be important to evaluate the potential impact of this activity profile in advance of using the pharmacoperones clinically.
